# Case report: Later onset of NRAS-mutant metastatic melanoma in a patient with a partially-excised giant congenital melanocytic nevus

**DOI:** 10.3389/fmed.2022.1086473

**Published:** 2022-12-08

**Authors:** Bruno Almeida Costa, Victor Zibara, Vasundhara Singh, Omid Hamid, Sonal Gandhi, Andrea P. Moy, Allison S. Betof Warner

**Affiliations:** ^1^Department of Medicine, Icahn School of Medicine at Mount Sinai (Morningside/West), New York, NY, United States; ^2^The Angeles Clinic and Research Institute, Cedar Sinai Affiliate, Los Angeles, CA, United States; ^3^Department of Pathology, Memorial Sloan Kettering Cancer Center, New York, NY, United States; ^4^Department of Medicine, Memorial Sloan Kettering Cancer Center, New York, NY, United States

**Keywords:** metastatic melanoma, congenital melanocytic nevi, giant nevus, *NRAS* mutation, MDM2 amplification

## Abstract

Despite recent advances in treatment and surveillance, metastatic melanoma still carries a poor prognosis. Large/giant congenital melanocytic nevi (CMNs) constitute a known risk factor for the condition, with the greatest risk for malignant transformation thought to be during childhood (median age at diagnosis of 3 years in a previous cohort). Herein, we present the case of a 30-year-old male who, after undergoing multiple excision/grafting procedures for a giant CMN as a child, was diagnosed with an *NRAS*-mutant, *MDM2*-amplified metastatic melanoma more than 20 years later. Response to ipilimumab/nivolumab immunotherapy, cisplatin/vinblastine/temozolomide chemotherapy, and nivolumab/relatlimab immunotherapy was poor. This case highlights the importance of lifetime monitoring with once-yearly dermatological examination (including lymph node palpation) in large/giant CMN patients, as well as the need for further clinical trials evaluating novel therapies for *NRAS*-mutant melanoma.

## Introduction

According to GLOBOCAN 2020, cutaneous melanoma accounts for 1.7% of new cancer cases worldwide (1). In the US, melanoma is now the fifth most commonly diagnosed malignancy, with around 99,780 cases estimated for 2022 (2). Over the last decade, US mortality decreased by nearly 30%, in part due to Food and Drug Administration (FDA) approval of several targeted and immune-based agents for patients with advanced disease. Even so, overall survival (OS) for stage IV melanoma remains low (29.8% at 5 years), prompting continuous bench-to-bedside efforts to develop novel therapies (3). Established risk factors for melanoma include a personal or family history of the malignancy, high socioeconomic status, Fitzpatrick skin phototype I-II, ultraviolet (UV) radiation by sun exposure or indoor tanning, and presence of acquired or congenital melanocytic nevi (CMNs) (4, 5).

With an estimated prevalence of 0.2–6% in worldwide literature, CMNs are benign proliferations of melanocytes often caused by postzygotic *NRAS* mutations *in utero* (6–9). Based on their projected adult size (PAS), these lesions can be classified as small (<1.5 cm), medium (1.5–20 cm), large (>20–40 cm), or giant (>40–60 cm) (9). CMNs >20 cm are uncommon, occurring in 1 out of every 20,000 births (8). The most frequent distribution pattern is bathing trunk (45.5%), followed by bolero (27.4%), back (13.6%), breast/belly (4.5%), body (4.5%), and body extremity (4.5%) (7). Additional descriptors include color heterogeneity, surface rugosity, presence of hypertrichosis, presence of dermal/subcutaneous nodules, and number of associated satellite lesions (9).

In a British cohort of 448 CMN patients aged 0-16 years, 10 (2.2%) developed melanoma, with a mean and median age at death from melanoma of 3.9 and 2.5 years, respectively. All 10 cases occurred in children with multiple CMNs, while 7 cases occurred in patients with a PAS >60 cm for the largest lesion (10). Nevertheless, melanoma risk estimates for patients with large or giant CMNs (LGCMNs) are imprecise, particularly due to significant biases of prior studies (e.g., relatively short length of follow-up, narrow age range for inclusion, or small sample sizes due to the disease's rarity) (11–14). Herein, we describe the case of a 30-year-old male with a childhood history of multiple excision/grafting procedures for a giant CMN who, more than 20 years later, developed an *NRAS*-mutant metastatic melanoma.

## Case presentation

A 30-year-old Caucasian male, previously healthy, presented to the emergency department (ED) complaining of intermittent upper back pain. It started the previous evening while he was running and had a moderate intensity, stabbing quality, and radiation to the right chest. The patient denied any similar prior episodes, specific aggravating/alleviating factors, or associated acute-onset symptoms. On review of systems, he described a 12-lb weight loss over 3 months, besides having noted a painless, slow-growing right axillary lump for the previous 2 months. There were no additional constitutional symptoms, swelling of other areas (such as neck, inguinal region, or testicles), or easy bleeding/bruising. He also negated prior thromboembolic events, medication/hormonal use, or recent trauma, surgery, travels, infections, or sick contacts.

Past medical history was significant for a “birthmark” extending over his abdomen and lower back, for which multiple excision/grafting procedures were performed at 6 years of age. He denied any itching, bleeding, or noticeable changes in the lesion's size, texture, color, or appearance for the last 20 years. The patient did not recall a specific diagnosis but reported consistent follow-up and mole mapping with a dermatologist outside the US. Despite being born in the UK, he lived in South Africa from early childhood until his 23 years of age and then returned to his home country, where he stayed until moving to the US a few months before presentation. When he was 10 years old, his father was treated for pulmonary tuberculosis. No other relevant family history was reported. He denied excess alcohol intake, current/former smoking, illicit drug use, overexposure to UV radiation, or known occupational hazards.

**Figure 1:**
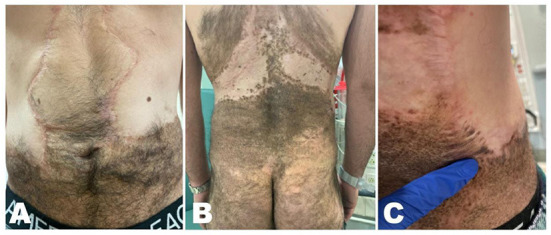
Heterogeneous brown-to-black patches and plaques with satellite lesions, areas of hypertrichosis and irregular borders, consistent with partially-excised giant congenital melanocytic nevus. **(A)** Scattered lesions over the lower abdomen. **(B)** Scattered lesions over the posterior thorax and buttocks. **(C)** Right mid-back nodular area where the initial incisional biopsy was performed.

During bedside evaluation, the patient was found to have heterogeneous brown-to-black patches/plaques scattered on his torso, buttocks, and lower abdomen with satellite lesions, areas of hypertrichosis, and irregular borders, consistent with partially-excised giant CMN of bathing trunk distribution (Figure 1). The skin lesions were mostly flat except for a nodular border where grafting was previously done. In addition, a 2-cm mobile, firm, non-tender and non-erythematous subcutaneous nodule was palpated in the right axillary region. His physical exam was otherwise unremarkable.

On initial workup, blood counts, basic chemistries, liver function tests, troponin levels, urinalysis, and electrocardiogram were within normal limits. Conversely, serum D-dimer was elevated (2.42 mcg/mL) and chest radiography showed a left lower lobe (LLL) density of approximately 3 cm, leading to the acquisition of thoracic computed tomography (CT) angiography. Despite a lack of pulmonary emboli, significant findings included two LLL nodules (2.7 and 1.0 cm), a left posterior pleural-based nodule (0.8 cm), and a right lower lobe nodule (0.3 cm).

The patient was admitted to the hospital for further diagnostic evaluation. While serum lactate dehydrogenase (LDH) was elevated (350 U/L), other laboratory tests resulted negative (including traditional tumor markers, hepatitis/HIV testing, QuantiFERON-TB Gold, and three sputum acid-fast bacillus smears). Contrast-enhanced CT (CECT) of the abdomen demonstrated hypodense lesions in the left and right adrenal glands (4.7 × 4.1 and 3.7 × 3.5 cm, respectively), between liver segments 2/3 (2.8 × 2.4 cm), and in the left inferior renal pole (1.4 × 1.1 cm). Brain magnetic resonance imaging (MRI) showed multiple enhancing parenchymal nodules of 0.4–1.2 cm, some of them with surrounding edema. Meanwhile, a whole spine MRI found no additional disease in the central nervous system (CNS) or vertebral bodies.

**Figure 2:**
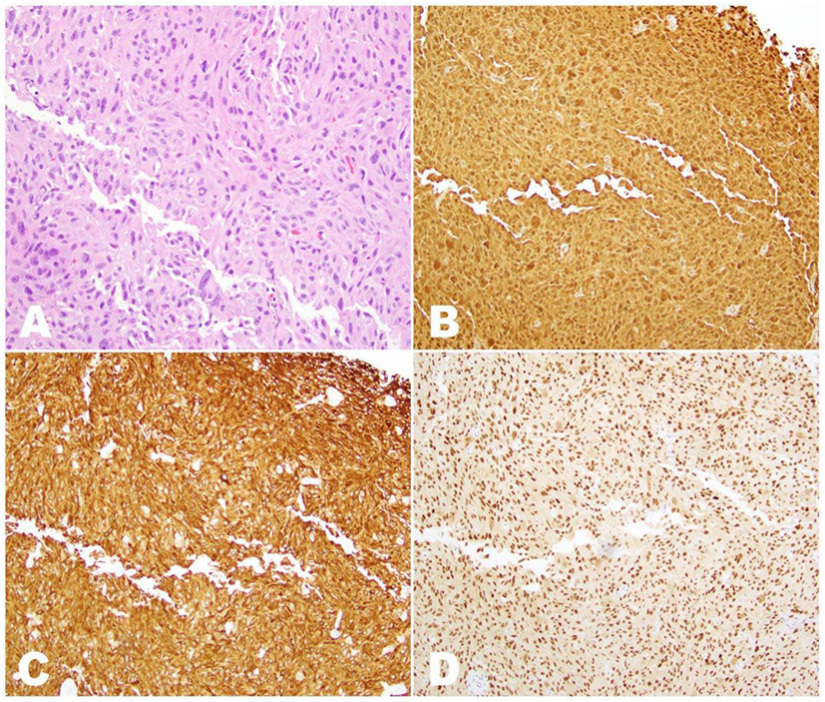
Histopathologic examination of the sample obtained by incisional biopsy of the right axillary nodule. **(A)** H&E stain (20 × magnification), showing innumerous irregularly-shaped tumor cells with nuclear hyperchromasia and prominent nucleoli intermixed within fibroadipose tissue. **(B)** Immunostaining for S100 (10 × magnification) showing diffuse nuclear and cytoplasmic positivity in all tumor cells. **(C)** Immunostaining for melan-A (10 × magnification) showing diffuse cytoplasmic positivity in all tumor cells. **(D)** Immunostaining for PRAME (10 × magnification) showing diffuse nuclear positivity in all tumor cells.

The high suspicion of metastatic cancer prompted an incisional biopsy of the right mid-back nodular area. Histopathological evaluation lacked evidence of malignancy and was consistent with reactive melanocytic proliferation to an underlying scar. As a result, the patient underwent an incisional biopsy of the right axillary nodule, with formalin-fixed paraffin-embedded (FFPE) samples revealing malignant cells within fibroadipose tissue (Figure 2). Immunohistochemistry (IHC) showed positivity for preferentially expressed antigen in melanoma (PRAME), S100, melan-A, and tyrosinase—a pattern consistent with melanoma—and negativity for *BRAF* V600E and *NRAS* Q61R. A hybridization capture-based next-generation sequencing assay (MSK-IMPACT) was also applied to the FFPE specimens. Although no microsatellite instability or structural variants were found, the tumor was positive for somatic mutations in *NRAS* exon 3 (c.181C>A, p.Q61K), *EP300* exon 31 (c.5992G>A, p.G1998R), and *MSH2* exon 12 (c.1996A>G, p.I666V). Additional findings included an estimated tumor mutation burden of 2.5 mutations/megabase and *MDM2*/*GLI1*/*ERBB3*/*CDK4*/*IGF1*/*TERT*/*SDHA*/*EP300*/*MSH2* amplification.

On account of his elevated serum LDH levels and CNS metastases, M1d(1) melanoma was ultimately diagnosed. As surgical metastasectomy was not appropriate, upfront treatment consisted of ipilimumab/nivolumab (3 mg/kg and 1 mg/kg, respectively, administered intravenously once every 3 weeks) and multifraction stereotactic radiosurgery of the brain (27 Gy divided into 3 daily fractions). Low-grade adverse events (hepatitis, thyroiditis, and oral mucositis) occurred after the first cycle of ipilimumab/nivolumab, but did not require immunotherapy discontinuation. Following 6 weeks of treatment, brain MRI showed interval contraction of CNS lesions and chest CTCE demonstrated stable pulmonary lesions. On the other hand, abdominopelvic imaging revealed new mesenteric/inguinal adenopathy and increased metastatic involvement of liver, kidneys, and adrenals.

**Figure 3:**
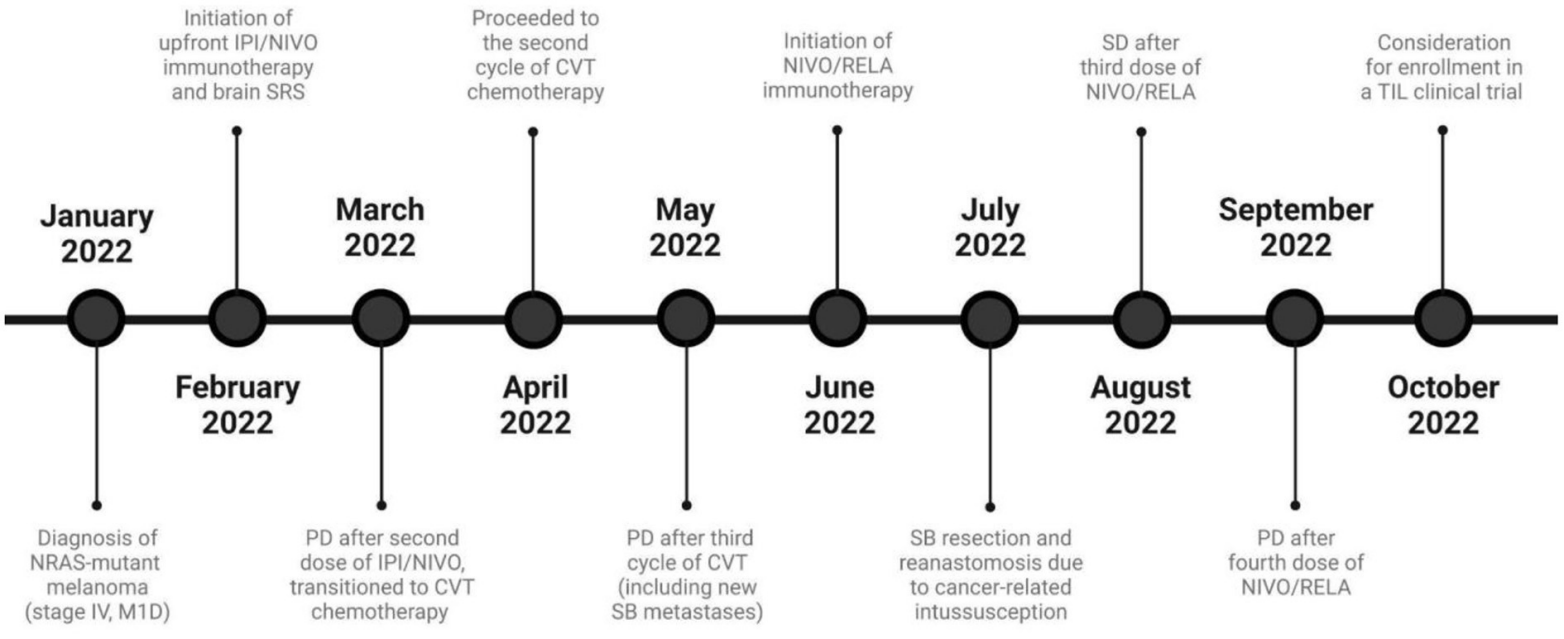
Timeline of the patient's clinical course and melanoma-directed therapy. CVT, Cisplatin/vinblastine/temozolomide; IPI, Ipilimumab; M1D, Metastasis to the central nervous system, with or without involvement of other sites; NIVO, Nivolumab; PD, Progressive disease; RELA, Relatlimab; SD, Stable disease; SB, Small bowel; TIL, Tumor-infiltrating lymphocytes.

The patient's rapid disease progression led to subsequent-line treatment with CVT (cisplatin 20 mg/m^2^ IV on days 1–4, vinblastine 1.6 mg/m^2^ IV on days 1–4, and temozolomide 150 mg/m^2^ orally on days 1–5 administered every 21 days). Following 3 cycles of cytotoxic chemotherapy, CECT disclosed nonobstructing transient small bowel intussusceptions secondary to intra- and extraluminal metastatic lesions. As a result, the patient was transitioned to nivolumab/relatlimab (480 mg/160 mg IV once every 4 weeks). A few days after the second combination dose, he presented to the ED complaining of intractable abdominal pain. Repeat abdominal CECT showed an edematous, hypoattenuating closed-loop small bowel obstruction with twisting of the mesentery. Given the evidence of significant ischemia, exploratory laparotomy with partial small bowel resection and reanastomosis was performed. The patient recovered well, albeit with considerable weight loss related to continued anorexia and abdominal pain. Stable disease (<20% growth of target lesions) was observed after three nivolumab/relatlimab doses. However, interval imaging after the fourth dose showed progression of the thoracic and abdominopelvic masses. Figure 3 showcases a timeline with relevant data from the patient's clinical course.

## Discussion

Progression to melanoma (cutaneous or extracutaneous) occurs in <1% of individuals with small or medium CMNs (10). In comparison, LGCMN patients have a 2.0–8.5% chance of malignant transformation (11, 12). Within this population, the probability of a lesion >20 cm evolving into cancer is not constant throughout life, with most evidence suggesting an increased risk during early childhood (11–14). Noteworthy, melanoma is otherwise uncommon in children (0.032% of cases occur in individuals age 10 or younger) (13). Among LGCMN patients who develop melanoma, 50% are diagnosed within the first 5 years of life, with another 20% of cases being detected before puberty (13, 14).

In the above-described case, a 30-year-old male with a childhood history of a giant CMN was diagnosed with metastatic melanoma more than 20 years after partial nevus excision. This presentation is potentially rare, as LGCMN patients seem to experience a substantial decrease in their melanoma risk following pubertal onset (14–16). For instance, in a retrospective cohort of 379 LGCMN patients from 26 countries, the median and mean age at melanoma diagnosis were 3 and 8 years, respectively (15). Therefore, the present report highlights the continued risk of malignant transformation during adulthood for this population. Correspondingly, a few other similar cases have been described in the literature, including adults up to 70 years of age who also had undergone partial excision of their lesions (17–20).

Due to the low incidence of LGCMNs, current evidence on the appropriate management of the condition is somewhat scarce. Some clinicians defend an observation-only approach, with close monitoring for any signs of malignant transformation (e.g., color/size changes or nodularity). In contrast, others consider early surgical excision crucial to prevent progression to melanoma (11–14). Entirely excising LGCMNs remains a challenging task—nevus cells often aggregate in the reticular dermis, subcutis, and subfascial layers (e.g., deeper muscle and nerve structures), making complete excision very complex and often impossible (18, 21). Furthermore, the benefit on preventing malignant transformation seems to be limited—in a retrospective review of 950 patients with cosmetically-challenging CMNs (age 1.8–19.2 years at the time of last evaluation), no patients developed melanoma within small residual lesions (13). By analyzing histopathological changes over time in 21 CMN patients, Gassenmaier et al. (22) suggested that the lesion's cellularity and pigment production decrease with age, the histological pattern and extension in depth remain stable, and clear resection margins are rarely attainable in larger lesions.

In recent years, a paradigm shift on the long-term care and modern surgical treatment of CMNs has emerged, establishing the long-term aesthetic outcomes at the center of any therapeutic endeavor. According to CMN Surgery Network recommendations, adequate counseling on conservative and/or surgical management requires an interdisciplinary exchange among physicians and individualized planning of the intervention, which frequently involves a multi-stage procedure. Treatment-related adverse effects (e.g., hospitalization, impaired wound healing, and hypertrophic scarring) must be carefully weighed against the prospects of a beneficial outcome—for instance, dermabrasion has been often associated with cosmetically unfavorable results and considerable repigmentation rates (23). Although melanoma prevention plays only a minor role in management, the risk of malignant transformation seems to persist throughout the patient's life. In this scenario, the above-cited tendency of nevus cells to develop deep in the subcutaneous tissue (as well as in the CNS in the setting of neurocutaneous melanosis) can hinder malignancy detection at earlier stages. Moreover, LGCMN-related melanomas have a greater propensity toward early metastases, as tumor cells are highly anaplastic (21). A reasonable way to deal with LGCMNs in adults could be lifetime monitoring with once-yearly dermatological examination (including lymph node palpation), despite a lack of prospective studies supporting this course of action (24, 25).

Further challenges are present after diagnosis, as molecular profiling varies widely (8). In a Chinese study, *BRAF* V600E mutations were not seen in LGCMNs, significantly contrasting with small and medium CMNs. Moreover, *BRAF* V600E never coexisted with *NRAS* exon 3 (codon 61) mutations in the same sample (26). In a Belgian series of 24 LGCMN patients, there was a high frequency of *NRAS* mutations (75% of cases) but *BRAF* mutations were less common (12% of cases) (27). Among the 19 LGCMN patients examined in a French study, 16 (84%) displayed an *NRAS* exon 3 (codon 61) mutation, while 1 carried a *BRAF* V600E mutation and 2 lacked alterations in those genes (28). In the present case, although IHC was negative to BRAF V600E and *NRAS* Q61R, subsequent molecular testing detected an *NRAS* Q61K mutation (seen in 34–50% of *NRAS*-mutant melanomas) (28, 29). Compared to other melanoma subtypes, *NRAS*-mutant tumors tend to be more aggressive and lead to worse outcomes (30, 31). For instance, our patient already had multiorgan metastases at the time of diagnosis, despite reporting few symptoms and no skin changes during initial evaluation.

Over the last decade, immune checkpoint inhibitors (ICIs) and targeted agents have significantly improved survival trends and response rates in BRAF-mutant melanoma. However, the ideal treatment for patients with *NRAS*-mutant melanoma remains unknown, especially due to the scarcity of prospective trials evaluating novel therapies in this patient subgroup (31, 32). Retrospective data has suggested that patients with *NRAS* mutations have higher response rates to immunotherapies, such as high-dose interleukin-2 and monoclonal antibodies (mAbs) against cytotoxic T-lymphocyte-associated protein 4 (CTLA4) or programmed cell death protein 1 (PD-1) (31–34). However, our patient responded poorly to first-line immunotherapy associating ipilimumab (anti-CTLA4 mAb) and nivolumab (anti-PD-1 mAb), developing rapid disease progression after 2 cycles. In the phase II Adaptively Dosed Immunotherapy (ADAPT-IT) trial, Postow et al. suggested that the efficacy of ipilimumab/nivolumab was driven by the first 2 combination doses, with patients being very unlikely to start responding after cycle 3 or 4 (35). From this perspective, our patient's treatment was switched to CVT, a multiagent cytotoxic regimen deemed well-tolerated and moderately efficacious in a phase II trial by the Hellenic Cooperative Oncology Group. In this study, subjects with BRAF-mutated tumors showed better response rates than those with BRAF wild-type tumors (39 vs. 27%), although subgroup analysis according to *NRAS* status was not performed (36). Ultimately, our patient's response to this line of therapy was poor. It is worth noting that little consensus exists regarding optimal standard chemotherapy for metastatic melanoma, which may reflect the low level of activity of older FDA-approved cytotoxic drugs and equivocal results from comparative phase III studies (37).

In March 2022, a fixed-dose combination of relatlimab—an anti-lymphocyte activation gene-3 (LAG-3) mAb—and nivolumab received FDA approval for advanced melanoma (38). This decision was based on the multinational, double-blinded, randomized phase II/III RELATIVITY-047 trial, which compared nivolumab/relatlimab vs. nivolumab monotherapy in 714 patients with newly-diagnosed metastatic or unresectable stage III/IV melanoma. After a median follow-up of 13.2 months, relatlimab's addition was associated with a significant increase in median progression-free survival (10.1 vs. 4.6 months; hazard ratio [HR], 0.75; 95% confidence interval [CI], 0.62–0.92; *P* = 0.006) (39). Correspondingly, the combined blockade of LAG-3 and PD-1 has been shown to promote synergistic effects in T-cell activation, causing enhanced antitumor activity compared to either alone (40).

MEK1/2 inhibition recently emerged as another therapeutic approach for *NRAS*-mutant melanoma (32, 37). In the phase III NEMO trial, binimetinib was associated with an overall response rate (ORR) of 15% and improved PFS compared with dacarbazine (2.8 vs. 1.5 months; HR, 0.62; 95% CI, 0.47–0.80; *P* < 0.001) (41). Thus, MEK inhibitors can be considered a useful option in patients with *NRAS*-mutant melanoma after failed immunotherapy. However, these agents are not widely available and further studies are needed to strengthen their incorporation into clinical practice (42). Although our patient had an MDM2 mutation detected, his CNS involvement deemed him ineligible for a clinical trial with an MDM2 inhibitor (NCT03611868) (43). Unfortunately, his molecular profiling did now show any additional targetable mutations that would allow management with other approved targeted drugs. Tumor-infiltrating lymphocyte (TIL) therapy as part of a clinical trial is a promising anti-melanoma strategy to be considered in patients with relapsed/refractory disease (44). In 2021, the phase 2 C-144-01 trial supported lifileucel's efficacy for advanced melanoma patients previously treated with ICIs and BRAF ± MEK targeted agents. Given the ORR of 36% (95% CI, 25–49) obtained with this agent (45), FDA approval is currently being sought. In addition to ongoing studies with lifileucel, novel TIL products are being actively investigated in multicentric trials (NCT05050006, NCT03997474) (44).

## Conclusion

This report highlights the importance of lifetime monitoring for progression to melanoma in large/giant CMN patients, regardless of whether partial/complete excision was performed. Although previous cohorts suggest that most malignant transformations occur during childhood, adults with a history of large/giant CMNs remain at a significantly higher risk of developing melanoma than the general population. As illustrated by the present case, melanomas associated with large/giant CMNs often harbor *NRAS* mutations—a biomarker of disease aggressiveness and worse clinical outcomes. Given that the ideal management for patients with *NRAS*-mutant melanoma remains unknown, further clinical studies are urgently needed to improve their prognosis.

## Data availability statement

The original contributions presented in the study are included in the article/supplementary material, further inquiries can be directed to the corresponding author/s.

## Ethics statement

Written informed consent was obtained from the individual(s) for the publication of any potentially identifiable images or data included in this article.

## Author contributions

BC, AB, VZ, and VS conceived and designed the study. BC, VZ, SG, AM, OH, and AB collected, analyzed, and interpreted clinical data. BC, VZ, SG, and AM wrote the first draft of the manuscript. VS, OH, and AB critically reviewed the manuscript for important intellectual content. All authors approved the final version of the manuscript.
